# Numerical study of unsteady tangent hyperbolic fuzzy hybrid nanofluid over an exponentially stretching surface

**DOI:** 10.1038/s41598-023-32374-1

**Published:** 2023-09-20

**Authors:** Muhammad Nadeem, Imran Siddique, Zakara Riaz, Basim M. Makhdoum, Rana Muhammad Zulqarnain, Mohammed Sallah

**Affiliations:** 1 Department of Mathematics, University of Management and Technology, Lahore, 54770 Pakistan; 2 Department of Mathematics, The University of Chenab, Gujrat, 50700 Pakistan; 3 Mechanical Engineering Department, College of Engineering and Islamic Architecture, Umm Al-Qura University, P. O. Box 5555, Makkah, 21955 Saudi Arabia; 4 Department of Mathematics, Zhejiang Normal University, Jinhua, 321004 Zhejiang China; 5 Applied Mathematical Physics Research Group, Physics Department, Faculty of Science, Mansoura University, Mansoura, 35516 Egypt; 6 Department of Physics, College of Sciences, University of Bisha, P.O. Box 344, Bisha 61922, Saudi Arabia

**Keywords:** Engineering, Nanoscience and technology, Physics

## Abstract

The significance of fuzzy volume percentage on the unsteady flow of MHD tangent hyperbolic fuzzy hybrid nanofluid towards an exponentially stretched surface is scrutinized. The heat transport mechanism is classified by Joule heating, nonlinear thermal radiation, boundary slippage, and convective circumstances. Ethylene glycol (EG) as a host fluid along with the nanomaterial’s Cu and ${\text{Al}}_{{2}} {\text{O}}_{{3}}$ are used for heat transfer analysis is also considered in this investigation. The nonlinear governing PDEs are meant to be converted into ODEs employing appropriate renovations. Then, a built-in MATLAB program bvp4c is employed to acquire the outcome of the given problem. The variation of flow rate, thermal heat, drag force and Nusselt number and their influence on fluid flow with heat transfer have been scrutinized through graphs. An increase in thermal radiation, power law index and nanoparticle volume friction heightens the heat transmission rate. Skin friction is diminished by swelling the power-law index, Weissenberg number, and ratio parameters, whereas it is increased by enhancing the magnetic parameter. The heat transfer rate upsurges with an increase in Weissenberg number and nanoparticle volume fraction. Also, the nanoparticle volume percentage is expressed as a triangular fuzzy number (TFN). The triangular membership function (MF) and TFN are regulated by the $\chi - {\text{cut}}$ parameter, which has a range of 0 to 1. In comparison to nanofluids, hybrid nanofluids have a higher heat transmission rate, according to the fuzzy analysis. This investigation has applications in the areas of paper manufacturing, metal sheet cooling and crystal growth.

## Introduction

The researchers have studied different types of non-Newtonian fluid (NNF) models for better flow behaviour in the area of engineering and science. A fluid that deviates from Newton's law of viscosity is recognized as a NNF. When forced, viscosities in NNF can change to become more liquid. In a NNF, the shear amount and shear stress correlate. The tangent hyperbolic liquid model is dynamic due to it can identify the shear-thinning and thickening phenomenon^1^. Also, tangent hyperbolic fluids are a form of NNF that belongs to the rate type fluids group and has equations in both high and mild tensile forces that are accommodated. Scientists suggest the Tangent hyperbolic fluid model due to its application in industrial and laboratory trials such as sauces, melted cheese, whipped cream, nail varnish, ketchup, and blood. Naseer et al.^2^ also evaluated the flow through the boundaries of tangent hyperbolic fluid across a vertically stretched cylinder. Using the homotopy analysis method (HAM), tangent hyperbolic motile microorganisms' nanomaterial flow was appraised by Shafiq et al.^3^. Hayat et al.^4^ reviewed MHD Tangent hyperbolic flow behaviour with variable thickness. Ibrahim and Gizewu^5^ detected tangent-hyperbolic fluid flow with dual dispersal mechanisms and a second-order slip barrier at the edge. Using an exponentially extending surface, Siddique et al.^6^ assessed the impact of Dufour and Soret on second-grade nanofluid flow in an unsteady MHD system. A boundary layer (BL) flow of MHD tangent hyperbolic fluid completed by an exponentially extending sheet has been developed by several philosophers^7–13^.

The behaviour of substantially conductive fluids in the complicity of a magnetic field is described by magneto-hydrodynamics (MHD). A transmitting fluid creates an electric current as it passes over a magnet force, which may be used to regulate fluid flow. When an engineer named Hartman and Lazarus^14^ constructed an electromagnetic pump in 1919, it broadened the field of MHD research. The researchable has demonstrated a keen interest in the flow problems of electrically conducting fluids, for instance, the provision of certain medications, electric power generation, cancer therapy, asthma treatment, and the building of generators that produce electricity. Also, it's useful in astrophysics, plasma, MHD pumps, and other fields. Sometimes electrically conducting fluids have extensive treatment in the metallurgy, optical switches, optical modulators, optical grating and extending of plastic sheets. Electromagnet hydrodynamics techniques are used to regulate the flow of fluids when electric fields are imposed. Lorentz force is introduced in electromagnet hydrodynamics when an electrically conducting fluid is affected by an orthogonal magnetic field. The MHD convective warmth switch glide of viscosity nanofluid throughout a squeezed sheet was investigated via means of Rashidi et al.^15^. The impacts of thermal conduction and radiation in MHD flow caused by a slandering surface were scrutinized by Reddy et al.^16^. Aziz^17^ used the Dufour and Soret reactions for heat and mass transport to investigate the consequences of thermal energy and MHD on a permeable convective surface. The presence of a uniform transverse magnetic area became explored via the means by Ashornejad et al.^18^, who addressed the hassles with the usage of the RK-four integration manufacturer and the MAPLE software's shot approach. Raptis^19^ looked at the steady MHD natural convection flow and stability of electrically controlled fluids passing through porous material surrounded by two infinite horizontal plates. Salhudin et al.^20^ operated to discover the chemical response of reactive kinds and combined convection houses of MHD Williamson nanofluid in a pouring media and the actuator is activated across a non-isothermal cone plate.

Flow associated with heat radiation has long piqued the researcher's curiosity. By expanding energy and molecular movement, thermal radiation affects molecule concentration and raises the temperature. Both theoretically and practically, the momentum and energy transport in BL flow throughout a stretched surface is extremely important. The reason for this is that these flows have a larger range of uses in industries including polymer technology, metallurgy, melt spinning, glass blowing, the production of plastic or rubber sheets, food processing, fibre spinning, continuous casting, etc. In addition, several technical processes-including involving the combustion of fossil fuels, solar energy, spacecraft re-entry and astrophysical flows-occur at higher temperatures. The strongly dependent radiation on an incompressible peristaltic flow via a stretched membrane was found by Pop et al.^21^. Due to heat radioactivity and an electric moment on MHD flow achieved by the use of a moving object perpendicular plates were considered by Sandeep and Sugunamma^22^. The process of heat radio waves, spinning, MHD, viscous dissipation movement, and chemical sensitivity over a thinned pad were evaluated by Reddy et al.^23^. Ahmad and Sarmah^24^ audited the effect of excess heat on a temporal MHD flow with mass transmission via an impetuously started infinite diagonal plate. In the impact of radiation and thermal diffusion, Ahmad^25^ inspected MHD flow through mass transfer and free convection of a viscous, impermeable, and liquid that releases electrons. By using the Laplace transform approach, he was able to find perfect answers for velocity, temperature, and concentration. Osman et al.^26^ outlined analytically the consequences of thermal rays and color reactions on irregular MHD BL flow in a ridged medium with a stove. In tangent hyperbolic nano liquid slowdown line flow, Hayat et al.^27^ assessed the repercussions of kinetic and non-linear infrared spectrum processes. The thermal radiation flow has also been studied by other researchers for a variety of situations and geometries^28–32^.

In recent decades, Experts have given careful consideration to the selection of enhanced heat transfer fluids. In industrial and technical applications, regular fluids (ethylene glycol, oil, and water) are not often conscripted because their thermal conductivity and heat transmission in the fluids are poor. To rectify this shortcoming, a single kind of nano-sized particle is used in the aforementioned fluids known as 'nanofluid'^33,34^. The attractive abilities of nanofluids have drawn researchers and scientists to seek unique routes in the modern day. Nanofluids have incredible heat transmission capabilities, much beyond those of ordinary liquids. Microelectronics, computer microchips, and fuel cells all integrate nanofluids to maximize their thermal act. Nanofluids can also be operated in a multitude of different contexts, such as heat exchangers, electronic machine cooling, reactors, biomedicine, conveyance, and transformer cooling. Oztop and Nada^35^ scrutinised the merits of using nanofluids immersed in a rectangular enclosure. In mechanical and web engineering, nanofluids are renowned as the most remarkable coolants. Bilal et al.^36^ taught MHD nanofluid flow driven by a cone. Two distinct nanoparticles are merged in the same host fluid to yield a hybrid nanofluid. With the advancement of technology, heat transmission has become the most pivotal step. However, as technology advances, a new kind of fluid used to transmit heat known as hybrid nanofluid stems as the insertion of nanofluids, which is conjured up by dispersing either two or more varieties of nano-sized particles having high thermal stability that are permanent into a base fluid. Chemical processes, manufacturing, transportation, thermal power generation, and many other applications and sectors requiring heat generation necessitate heat transfer performance to get the best accuracy. Jana et al.^37^ and Turcu et al.^38^ were identified as the first to use hybrid microparticles in their field trials. The benefits of the $\left( {\text{Cu}} -{\text{Al}}_{{2}} {\text{O}}_{{3}} \right)$ hybrid nanoparticle were highlighted by Suresh et al.^39^, while Devi and Devi^40^ proposed correlations of thermal features for hybrid nanofluids in the analysis of a BL flow problem. Additionally, Takabi and Salehi^41^ projected correlations for hybrid nanofluids, which are frequently employed in the numerical simulation of fluid motion. In such a variety of geometries, various investigators utilized hybrid nanofluids^42–49^.

Different types of fuzziness or uncertainties occur in dynamical systems, and they are connected to error measurement, engineering parameters, dimensional tolerances, environmental influences, partial information, comparison, starting, material qualities, boundary conditions, and so on. Fuzziness or ambiguity will very certainly change the dynamic systems, perhaps influencing the result. These are not measured exactly, and their nominal values are not given. As a result of missing, imprecise or erroneous information, these values are foggy or uncertain in practice. In this position, using fuzzy sets theory (FST) is a more appropriate description of the phenomenon rather than presumptuous physical complications. To be more particular, FDEs are useful for reducing ambivalence and explaining bodily problems that arise when heat transmission constraints, beginning circumstances, and initial or boundary conditions are fuzzy. In 1965, Zadeh^50^ was the first to express the FST. FST is a beneficial tool for sharing circumstances where information is vague, confusing, or imprecise. The objective of membership or belongingness perceives FST. Each component of the discourse universe is given a number from the [0, 1] range by the MF in the FST. Fuzzy differentiability was initially brought up by Seikala^51^. Kaleva debated derivative and integration with fuzziness in^52^. Buckley and Feuring^53^ exploited FDEs to solve the nth-order DE using fuzzy initial conditions. Moreover, various scholars have executed FST to create well-known scientific and technical results^54–57^.

The investigations stated above show no struggle has been made to examine the unsteady tangent hyperbolic hybrid nanofluid flow across an exponentially stretched sheet. Through the use of suitable variables, nonlinear ODEs are produced. To create convergent solutions, the Bvp4c scheme is used. The originality of the work is as follows:

- The injection of magnetic flux into the region's flow is critical for managing the dynamic behaviour of the manufacturing process.
- The heat equation comprises nonlinear thermal radiation, joule heating, dissipation effects and heat source.
- Fuzzy solutions are designed to assess the empirical uncertain dispersion the nanoparticles volume fractions are regarded as triangle fuzzy numbers using the $\chi {\text{ - cut}}$ technique, and the fuzzy triangular MFs are used to define $\chi {\text{ - cut}}{.}$
- Fuzzy triangular MFs were utilized to compare the nanofluids and hybrid nanofluids.

## Formulation

**Figure 1:**
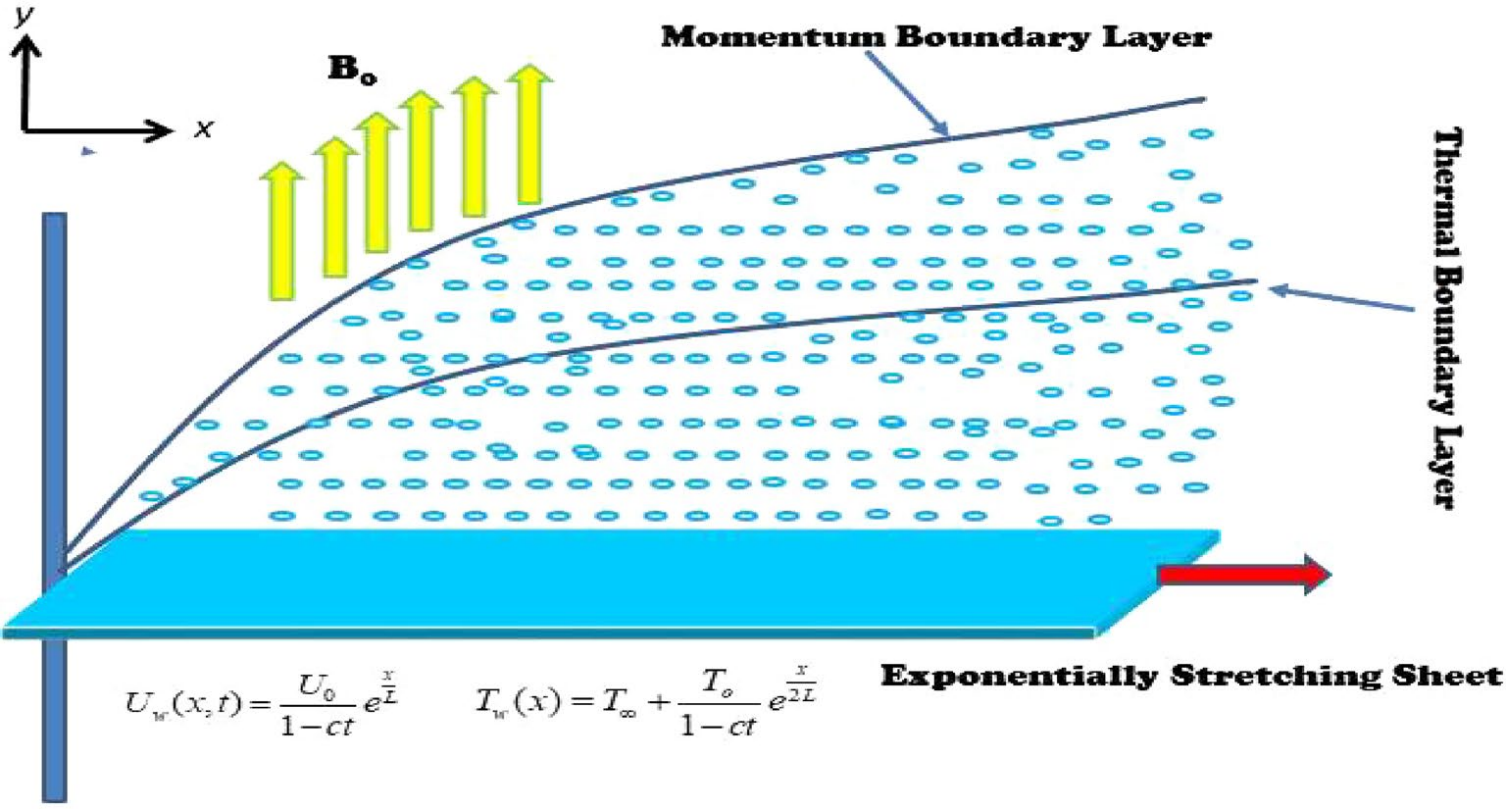
Flow geometry.

The flow of the equilibrium area of an unsteady tangent hyperbolic fuzzy hybrid nanofluid approaching a convectively warm exponentially stretchy sheet is pursued in two dimensions (2D). The *x*-axis is perpendicular to the stretching plane, whereas the *y*-axis is becoming longer upright to the *x*-axis. A viscous fluid's flow is constrained by $y > 0.$ The benefit of a non-uniform magnetic field, i.e.$B\left( x \right) = B_{ \circ } {\text{e}}^{x/2L}$ this is carried out in the opposite direction of the flow field shown in Fig. 1. The lack of a magnetic force that is triggered is due to the modest Reynolds number. It is presumed that the electric field is zero. The Joule heating ramifications are preserved. After the following assumptions, provided equations underlie BL flow and heat transport are given as^3,4^,

1 $$\frac{\partial u}{{\partial x}} + \frac{\partial v}{{\partial y}} = 0,$$

2 $$\frac{\partial u}{{\partial t}} + u\frac{\partial u}{{\partial x}} + v\frac{\partial u}{{\partial y}} = \frac{{\mu_{hnf} }}{{\rho_{hnf} }}\left[ {\left( {1 - \alpha } \right)\frac{{\partial^{2} u}}{{\partial y^{2} }} + \sqrt 2 \alpha \Gamma \left( {\frac{\partial u}{{\partial y}}} \right)\left( {\frac{{\partial^{2} u}}{{\partial y^{2} }}} \right)} \right] - \frac{{\delta_{hnf} B_{0}^{2} u}}{{\rho_{hnf} }},$$

3 $$\begin{aligned} & \frac{\partial T}{{\partial t}} + v\frac{\partial T}{{\partial y}} + u\frac{\partial T}{{\partial x}} = \frac{{k_{hnf} }}{{\left( {\rho C_{p} } \right)_{hnf} }}\frac{{\partial^{2} T}}{{\partial y^{2} }} + \frac{{\delta_{hnf} B_{ \circ }^{2} }}{{\left( {\rho C_{p} } \right)_{hnf} }}u^{2} + \frac{{Q_{ \circ } \left( {T - T_{\infty } } \right)}}{{\left( {\rho C_{p} } \right)_{hnf} }} - \frac{1}{{\left( {\rho C_{p} } \right)_{hnf} }}\frac{{\partial q_{r} }}{\partial y} \\ & \;\;\; + \frac{{v_{f} }}{{\left( {\rho C_{p} } \right)_{hnf} }}\left( {1 - \alpha } \right)\left( {\frac{\partial u}{{\partial y}}} \right)^{2} + \frac{{v_{f} \alpha \Gamma }}{{\left( {C_{p} } \right)_{hnf} \sqrt 2 }}\frac{\partial u}{{\partial y}}\left( {\frac{\partial u}{{\partial y}}} \right)^{2} , \\ \end{aligned}$$

boundary conditions (BCs) are:

4 $$\left. \begin{array}{ll} t < 0:\,\,\,\,T = T_{\infty } ,\,\,\,\,v = 0,\,\,\,\,u = 0,\,\,\,\,\,\,\,\,\forall \,x,\,y, \hfill \\ t \ge 0:\,\,\,\,v\left( {x,\,0} \right) = v_{w} ,\,\,\,\,u\left( {x,\,0} \right) = \mu_{hnf} \frac{\partial u}{{\partial y}} + U_{w} ,\,\,\,\,h_{f} \left( {T - T_{w} } \right) = - k_{hnf} \frac{\partial T}{{\partial y}},\,\,\,\,\,{\text{at}}\,\,y \to 0, \hfill \\ \,\,\,\,\,\,\,\,\,\,\,\,\,\,u \to 0,\,\,\,T \to T_{\infty } ,\,\,\,{\text{at}}\,\,y \to \infty . \hfill \\ \end{array} \right\}$$

The *x*-axis and *y*-axis velocity coefficients are symbolized by *u* and *v*, respectively. $$\sigma_{hnf}$$ is the electrical conductivity, $$\sigma^{ * }$$ the Stefan-Boltzmann constant, $$T_{\infty }$$ is the reference temperature, $$k_{hnf}$$ is the thermal conductivity, $$T$$ is the fluid temperature, $$\Gamma$$ the material constant, $$\rho_{hnf}$$ represents the density, $$\nu_{hnf}$$ kinematic viscosity, $$U_{ \circ }$$ is the reference velocity, $$\left( {c_{p} } \right)_{hnf}$$ represents the specific heat of hybrid nanofluid, $$T_{w}$$ the temperature of the convective fluid under the sheet, $$K^{ * }$$ the ratio of mean assimilation and $$h_{f} = h{\text{e}}^{{{\raise0.7ex\hbox{$x$} \!\mathord{\left/ {\vphantom {x {2L}}}\right.\kern-0pt} \!\lower0.7ex\hbox{${2L}$}}}}$$ the index of convective heat transfer. Table 1 lists the thermophysical features of a hybrid nanofluid.

**Table 1:** The ${\text{Cu}}$ thermo-physical properties along with ${\text{Al}}_{{2}} {\text{O}}_{{3}}$ and EG^41–43^.

Physical properties	$\rho \;\left( {{\text{kg/m}}^{3} } \right)$	$C_{p} \;\left( {\text{J/kgK}} \right)$	$k\;\left( {\text{W/mK}} \right)$	$\sigma \left( {\Omega /m} \right)^{ - 1}$
Ethylene Golcol (EG)	1114	2415	0.252	$5.5 \times 10^{ - 6}$
${\text{Al}}_{{2}} {\text{O}}_{{3}}$	3970	765	40	$3.69 \times 10^{7}$
${\text{Cu}}$	8933	385	401	$5.96 \times 10^{7}$

The following set of similarity transformations is used in Eqs. (1)–(3) and their related BCs, we must first transform them into non-dimensional ODEs by adding the following analogous variables:

5 $$\left. \begin{array}{ll} u = \frac{{ae^{{{\raise0.7ex\hbox{$x$} \!\mathord{\left/ {\vphantom {x L}}\right.\kern-0pt} \!\lower0.7ex\hbox{$L$}}}} f^{\prime}}}{1 - ct},\,\,\,v = - \sqrt {\frac{{av_{f} }}{{2L\left( {1 - ct} \right)}}} e^{{{\raise0.7ex\hbox{$x$} \!\mathord{\left/ {\vphantom {x {2L}}}\right.\kern-0pt} \!\lower0.7ex\hbox{${2L}$}}}} \left( {f\left( \eta \right) + \eta f^{\prime}\left( \eta \right)} \right),\,\,\, \hfill \\ \eta = \sqrt {\frac{a}{2\nu L(1 - ct)}} e^{{\left( {{\raise0.7ex\hbox{$x$} \!\mathord{\left/ {\vphantom {x {2L}}}\right.\kern-0pt} \!\lower0.7ex\hbox{${2L}$}}} \right)}} y,\,\,\,\,\,\theta \left( \eta \right) = \frac{{T - T_{\infty } }}{{T_{w} - T_{\infty } }}. \hfill \\ \end{array} \right\}$$

By applying Eq. (5), the continuity equation is satisfied and Eqs. (1)–(3) are

6 $$\frac{{\mu_{r} }}{{\rho_{r} }}(1 - \alpha )f^{\prime\prime\prime} + \frac{{\alpha \mu_{r} }}{{\rho_{r} }}f^{\prime\prime}f^{\prime\prime\prime}We - M\frac{{\delta_{r} }}{{\rho_{r} }}f^{\prime} - Bf^{\prime} - \frac{{\eta Bf^{\prime\prime}}}{2} - 2f^{{\prime}{2}} + ff^{\prime\prime} = 0,\,\,$$

7 $$\left. \begin{array}{ll} \beta \eta \theta^{\prime} - \theta^{\prime}f - \frac{{k_{r} \theta^{\prime\prime}}}{{(\rho C_{\rho } )_{r} P_{r} }} + \frac{{N_{r} \theta^{\prime\prime}}}{{(\rho C_{\rho } )_{r} P_{r} }}\left[ {1 + \theta \left( {\theta_{w} - 1} \right)} \right]^{3} - \frac{{3N_{r} \theta^{{\prime}{2}} \left( {\theta_{w} - 1} \right)}}{{(\rho C_{\rho } )_{r} P_{r} }}\left[ {1 + \theta \left( {\theta_{w} - 1} \right)} \right]^{2} \hfill \\ - \frac{{\delta_{r} }}{{(\rho C_{\rho } )_{r} }}MEcf^{{\prime}{2}} - \frac{H\theta }{{(\rho C_{\rho } )_{r} }} - \frac{(1 - \alpha )}{{(C_{\rho } )_{r} }}Ecf^{{\prime\prime}{2}} - \frac{\alpha }{{(C_{\rho } )_{r} }}\frac{EcWe}{2}\left( {f^{\prime\prime}} \right)^{3} = 0. \hfill \\ \end{array} \right\}$$

8 $$\left. \begin{array}{ll} f\left( \eta \right) = 0,\,\,f^{\prime}\left( \eta \right) = 1 + \gamma \mu_{r} f^{\prime\prime}\left( \eta \right),\,\,k_{r} \theta^{\prime}\left( \eta \right) = - B_{i} \left( {1 - \theta \left( \eta \right)} \right),\,\,\,\,{\text{at}}\,\,\,\eta \to 0, \hfill \\ f^{\prime}\left( \eta \right) = 0,\,\,\,\,\theta \left( \eta \right) = 0,\,\,{\text{at}}\,\,\,\eta \to \infty , \hfill \\ \end{array} \right\}$$

$$\begin{aligned} P_{r} & = \frac{{v_{f} }}{{\alpha_{f} }},\,\,\,\,\,\gamma = \frac{h}{K}\sqrt {\frac{{2v_{f} L}}{U \circ }} ,\,\,\,\,\,We = \frac{{\sqrt 2 U_{ \circ }^{{{\raise0.7ex\hbox{$3$} \!\mathord{\left/ {\vphantom {3 2}}\right.\kern-0pt} \!\lower0.7ex\hbox{$2$}}}} \Gamma }}{{\sqrt {v_{f} L} }},\,\,M = \frac{{2\sigma_{f} B_{ \circ }^{2} }}{{\rho_{f} U_{ \circ } }},\,\,\,N_{r} = \frac{{4\sigma^{ * } T_{\infty }^{3} }}{{K^{ * } k_{f} }},\,\,\,B_{i} = \frac{{h_{f} }}{{k_{f} }}\sqrt {\frac{{v_{f} }}{a}} ,\,\, \\ Ec & = \frac{{U_{w}^{2} }}{{\left( {Cp} \right)_{f} \left( {T_{w} - T_{\infty } } \right)}}, \\ \end{aligned}$$

here $\beta$ is unsteady parameter, $We$ is wessingbrg number, $B_{i}$ is Biot number, $M$ is magnetic parameter, $Ec$ is Eckert number, $\alpha$ is power law index, $\gamma$ is slip parameter, the radiation parameter $N_{r}$ and $\theta_{w}$ is a temperature difference. The thermophysical features of hybrid nanofluids are illustrated by Eq. (13).

The skin friction coefficient $\left( {Cf_{x} } \right)$ and local Nusselt number $\left( {Nu_{x} } \right)$ are offered by

9 $$C_{f} = \frac{{\tau_{w} }}{{\rho_{f} U_{w}^{2} }},\,\,\,Nu_{x} = \frac{{xq_{w} }}{{k_{f} \left( {T_{f} - T_{\infty } } \right)}},$$

where

10 $$\tau_{w} = \left[ {\left( {1 - \alpha } \right)\frac{\partial u}{{\partial y}} + \frac{\alpha \Gamma }{{\sqrt 2 }}\left( {\frac{\partial u}{{\partial y}}} \right)^{2} } \right]_{y = 0} ,\;\;\;q_{w} = - \left( {k_{hnf} + \frac{{16\sigma^{ * } T_{\infty }^{3} }}{{3K^{ * } }}} \right)\left( {\frac{\partial T}{{\partial y}}} \right)_{y = 0} .$$

In respect of dimensionless from one's point of view,

11 $$C_{f} {\text{Re}}_{x}^{{{\raise0.7ex\hbox{$1$} \!\mathord{\left/ {\vphantom {1 2}}\right.\kern-0pt} \!\lower0.7ex\hbox{$2$}}}} = \left( {\left( {1 - \alpha } \right)f^{\prime\prime}\left( 0 \right) + \frac{\alpha }{2}We\left( {f^{\prime\prime}\left( 0 \right)} \right)^{2} } \right),$$

12 $$\left( {{\text{Re}}_{x} } \right)^{ - 0.5} Nu_{x} = - \left( {k_{r} + N_{r} \left( {1 + \theta \left( 0 \right)\left( {\theta_{w} - 1} \right)} \right)^{3} } \right)\theta^{\prime}\left( 0 \right),$$

where ${\text{Re}}_{x} = \frac{{xU_{w} }}{\nu }$ symbolizes the local Reynolds number.

13 $$\left. \begin{array}{ll} \rho_{r} = \frac{{\rho_{hnf} }}{{\rho_{f} }} = \left[ {\left( {1 - \phi_{2} } \right)\left\{ {\left( {1 - \phi_{1} } \right) + \frac{{\rho_{{s_{1} }} \phi_{1} }}{{\rho_{f} }}} \right\} + \frac{{\rho_{{s_{2} }} \phi_{2} }}{{\rho_{f} }}} \right],\,\,\,\,\,\,\mu_{r} = \frac{{\mu_{hnf} }}{{\mu_{f} }} = \left( {1 - \phi_{1} } \right)^{ - 2.5} \left( {1 - \phi_{2} } \right)^{ - 2.5} , \hfill \\ \left( {\rho C_{\rho } } \right)_{r} = \frac{{\left( {\rho C_{\rho } } \right)_{hnf} }}{{\left( {\rho C_{\rho } } \right)_{f} }} = \frac{{\phi_{2} \left( {\rho C_{\rho } } \right)_{{s_{2} }} }}{{\left( {\rho C_{\rho } } \right)_{f} }} + \left( {1 - \phi_{2} } \right)\left[ {\left( {1 - \phi_{1} } \right) + \frac{{\left( {\rho C_{\rho } } \right)_{{s_{1} }} \phi_{1} }}{{\left( {\rho C_{\rho } } \right)_{f} }}} \right], \hfill \\ k_{r} = \frac{{k_{hnf} }}{{k_{nf} }} = \frac{{2k_{nf} - 2\phi_{1} \left( {k_{{s_{1} }} - k_{nf} } \right) + k_{{s_{1} }} }}{{2k_{nf} + \phi_{1} \left( {k_{{s_{1} }} - k_{nf} } \right) + k_{{s_{1} }} }},\,\,\,\,\,\,\frac{{k_{nf} }}{{k_{f} }} = \frac{{2k_{f} - 2\phi_{2} \left( {k_{{s_{2} }} - k_{f} } \right) + k_{{s_{2} }} }}{{2k_{f} + \phi_{2} \left( {k_{{s_{2} }} - k_{f} } \right) + k_{{s_{2} }} }}. \hfill \\ \end{array} \right\}$$

### Fuzzification

A change in the volume concentration value can affect the velocity and temperature profiles of a nanofluid and hybrid nanofluid in practice. Moreover, to observe the present situation, the nanoparticle volume fraction is regarded as a fuzzified parameter in aspects of a TFN (see Table 2). The $\chi {\text{ - cut}}$ technique is utilized to transfer the constitutional ODEs into FDEs. See the literature for more details about this topic^53–56^.

**Table 2:** TFN of *ϕ*1 and *ϕ*2^53–57^.

Fuzzy numbers	Crisp value	TFN	$\chi {\text{ - cut}}\,\,{\text{approch}}$
$\phi_{1} \,\,{\text{(Al}}_{{2}} {\text{O}}_{{3}} {)}$	[0.01–0.04]	[0, 0.05, 0.1]	$[0.05\chi ,\,0.1 - 0.05\chi ],\,\,\chi \in [0,1]$
$\phi_{2} \,\,\,{\text{(Cu)}}$	[0.01–0.04]	[0, 0.05, 0.1]	$[0.05\chi ,\,0.1 - 0.05\chi ],\,\,\chi \in [0,1]$

**Figure 2:**
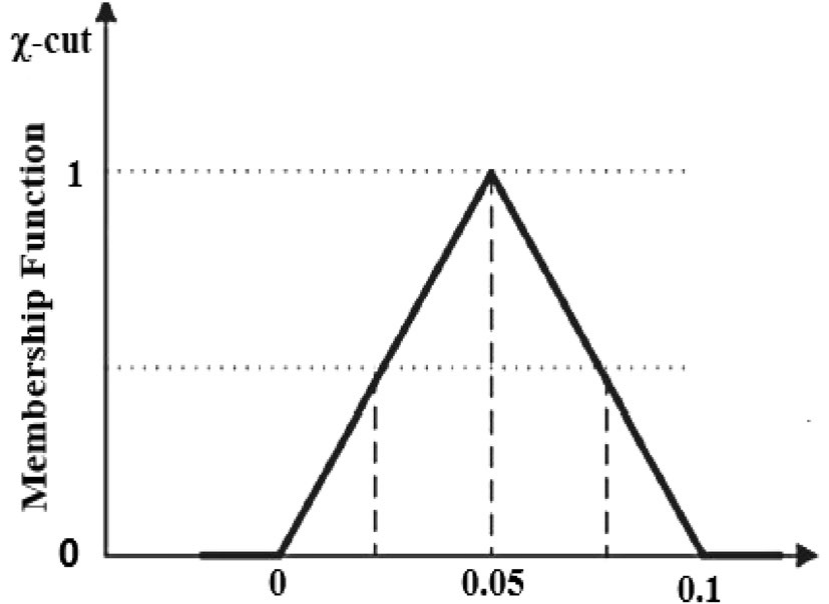
Membership function of TFN.

Let $\phi =$[0, 0.05, 0.1] be a TFN explained essentially by three measures: 0 (lower bound), 0.05 (most perception value), and 0.1 (upper bound) as shown in Fig. 2. By the TFN, the Membership function $M(\phi )$ can be expressed as

$$M\left( \phi \right) = \,\left\{ \begin{array}{ll} \frac{0 - \eta }{{0.05 - 0}}&\quad {\text{for }}\,\,\,\eta \in {[0,}\,\,{0}{{.05],}} \hfill \\ \frac{\eta - 0.1}{{0.1 - 0.05}}&\quad {\text{for}}\,\,\,\,\eta \in {[0}{{.05,}}\,\,{0}{\text{.1],}} \hfill \\{0}&\quad ,{\text{otherwise}}{.} \hfill \\ \end{array} \right.$$

TFNs are transformed into interval numbers employing the χ—cut method, which is indicated as $[\theta_{1} (\eta ,\,\,\chi ),\theta_{2} (\eta ,\,\,\chi )] = \overline{\theta } (\eta ,\chi ) = [0.05\chi ,\,\,\,0.1 - 0.05\chi ],\,\,\,{\text{where}}\,\,\chi {\text{ - cut}} \in {[0,}\,\,{1]}.$ The FDEs are changed into lower $\theta_{1} (\eta ,\,\,\chi )\,\,{\text{and}}\,\,{\text{upper}}\,\,{\text{bounds}}\,\,\,\,\theta_{2} (\eta ,\,\,\chi ).$

### Solution methodology

The differential system given above has nonlinear equations, making it impossible to determine an exact solution. So we shall use a numerical technique namely the bvp4c method. To use this algorithm, convert the set of non-linear ODEs and their BCs to a system of first-order ODEs and beginning assumptions.

14 $$\left. \begin{array}{ll} f = y_{1} \,\,\,\,\,f^{\prime} = y^{\prime}_{1} = y_{2} \,\,\,\,\,f^{\prime\prime} = y^{\prime}_{2} = y_{3} \,\,\,f^{\prime\prime\prime} = y^{\prime}_{3} \hfill \\ \theta = y_{4} \,\,\,\,\,\theta^{\prime} = y^{\prime}_{4} = y_{5} \,\,\,\,\,\theta^{\prime\prime} = y^{\prime}_{5} \hfill \\ \end{array} \right\}$$

15 $$y^{\prime}\left( 3 \right) = f^{\prime\prime\prime} = \frac{1}{{\frac{{\mu_{r} }}{{\rho_{r} }}(1 - \alpha ) + \eta \frac{{\mu_{r} }}{{\rho_{r} }}We(y(3))}}\left[ \begin{gathered} \frac{{\delta_{r} }}{{\rho_{r} }}My(2) + \beta y(2) + \frac{\eta \beta }{2}y(3) \hfill \\ + 2(y(2))^{2} - y(1)y(3) \hfill \\ \end{gathered} \right],$$

16 $$\theta^{\prime\prime} = y^{\prime}(5) = \frac{{\left( {\rho C_{\rho } } \right)_{r} \rho_{r} }}{{K_{r} + N_{r} \left\{ {1 + y(4)(\theta_{w} - 1)} \right\}^{3} }}\left[ \begin{gathered} \frac{{ - 3N_{r} (y(5))^{2} }}{{\left( {\rho C_{\rho } } \right)_{r} \rho_{r} }}(\theta_{w} - 1)\left\{ {1 + y(4)(\theta_{w} - 1)} \right\}^{2} + \beta \eta y(5) \hfill \\ - y(5)y(1) - \frac{{\delta_{r} }}{{\left( {\rho C_{\rho } } \right)_{r} }}MEc(y(2))^{2} - \frac{Hy(4)}{{\left( {\rho C_{\rho } } \right)_{r} }} \hfill \\ - \frac{(1 - \alpha )}{{(C_{\rho } )_{r} }}Ec(y(3))^{2} - \frac{\eta EcWe}{{(C_{\rho } )_{r} }}(y(3))^{3} \hfill \\ \end{gathered} \right],$$

17 $$\left. \begin{array}{ll} y_{1} = 0,\,\,\,\,y_{2} = 1 + \mu_{hnf} \gamma y(3),\,\,\,\,y_{5} = \frac{{B_{i} }}{{K_{hnf} /K_{f} }}(y_{4} - 1),\,\,\,\,\,{\text{at}}\,\,\,\eta = 0, \hfill \\ y_{2} \to 0,\,\,\,\,y_{4} \to 0,\,\,\,\,\,\,{\text{at}}\,\,\,\eta \to \infty . \hfill \\ \end{array} \right\}$$

For our specific problem, we are taking $10^{ - 5}$ as tolerance of error.

## Results and discussion

We employed two distinct types of $${\text{Al}}_{{2}} {\text{O}}_{{3}} \,\,{\text{and}}\,\,{\text{Cu}}$$ nanoparticles in base fluid EG that flow across an exponentially expanding surface for this research work. Also, this section's big priority is to discuss the habits of numerous parameters, such as the radiation parameter $$\left( {N_{r} = 0.6} \right)$$, unsteady parameter $$\left( {\beta = 0.5} \right),$$ the Prandtl number $$\left( {{\text{P}}_{r} = 7} \right)$$, magnetic parameter $$\left( {M = 0.3} \right),$$ the Biot number $$\left( {Bi = 0.2} \right)$$, the power law index the $$\left( {\alpha = 0.3} \right),$$$$\left( {Ec = 0.7} \right)$$ Eckert number, the temperature ratio $$\left( {\theta_{w} = 1.2} \right)$$ and nanoparticle consentration $$\left( {\phi_{1} = \phi_{2} = 0.02} \right).$$

The comparison is shown in Table 3 with an impressive accuracy level. As a result, it is expected that the results illustrated by the current numerical approach are extremely precise.

**Table 3:** Comparison of numerical values of $- f^{\prime\prime}\left( 0 \right)$ for $\beta$ when $\alpha = We = M = 0.$

$\beta$	$- f^{\prime\prime}\left( 0 \right)$ Mustafa et al.^13^	$- f^{\prime\prime}\left( 0 \right)$ Present
0	− 1.281809	− 1.281809
0.1	− 1.253580	− 1.253580
0.2	− 1.195120	− 1.195118
0.5	− 0.879835	0.879833
0.8	− 0.397771	0.397767
1.2	0.451571	0.451568

**Figure 3:**
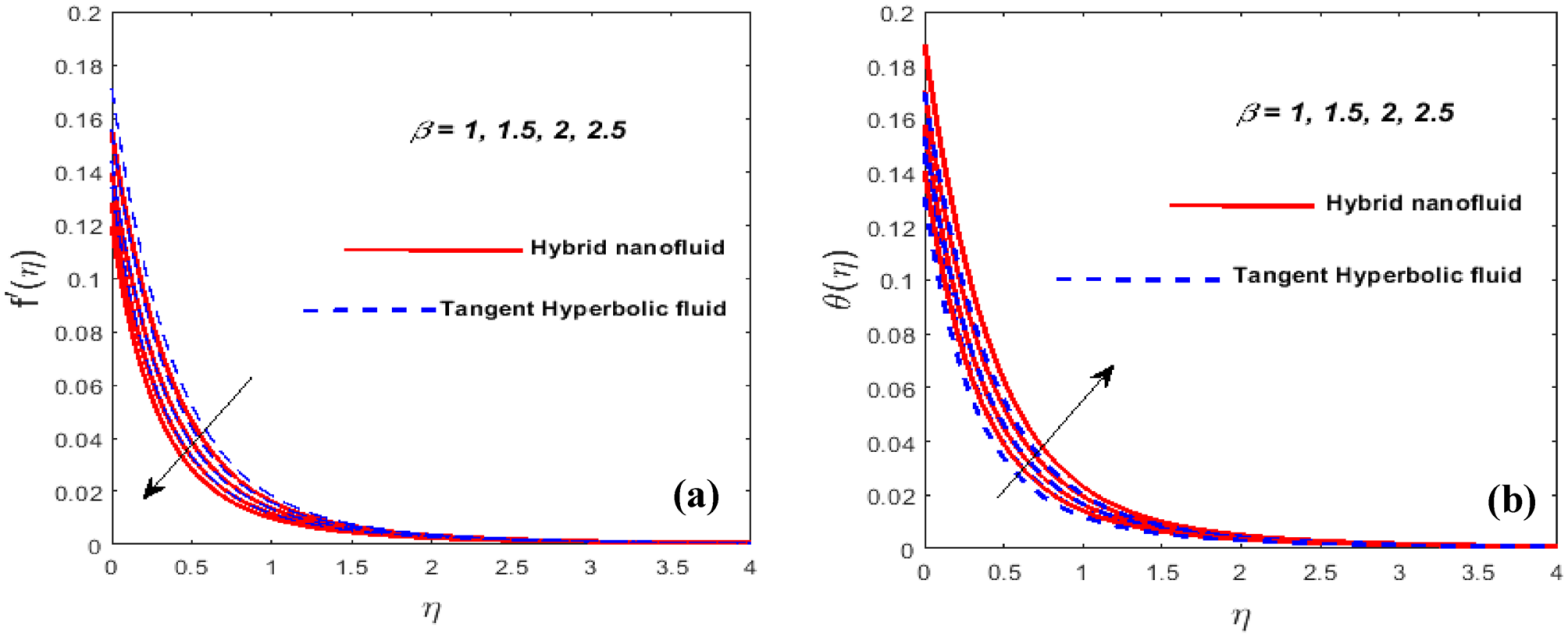
$$\beta$$ against $$f^{\prime}\left( \eta \right)$$ (**a**) and $$\theta \left( \eta \right)$$ (**b**).

**Figure 4:**
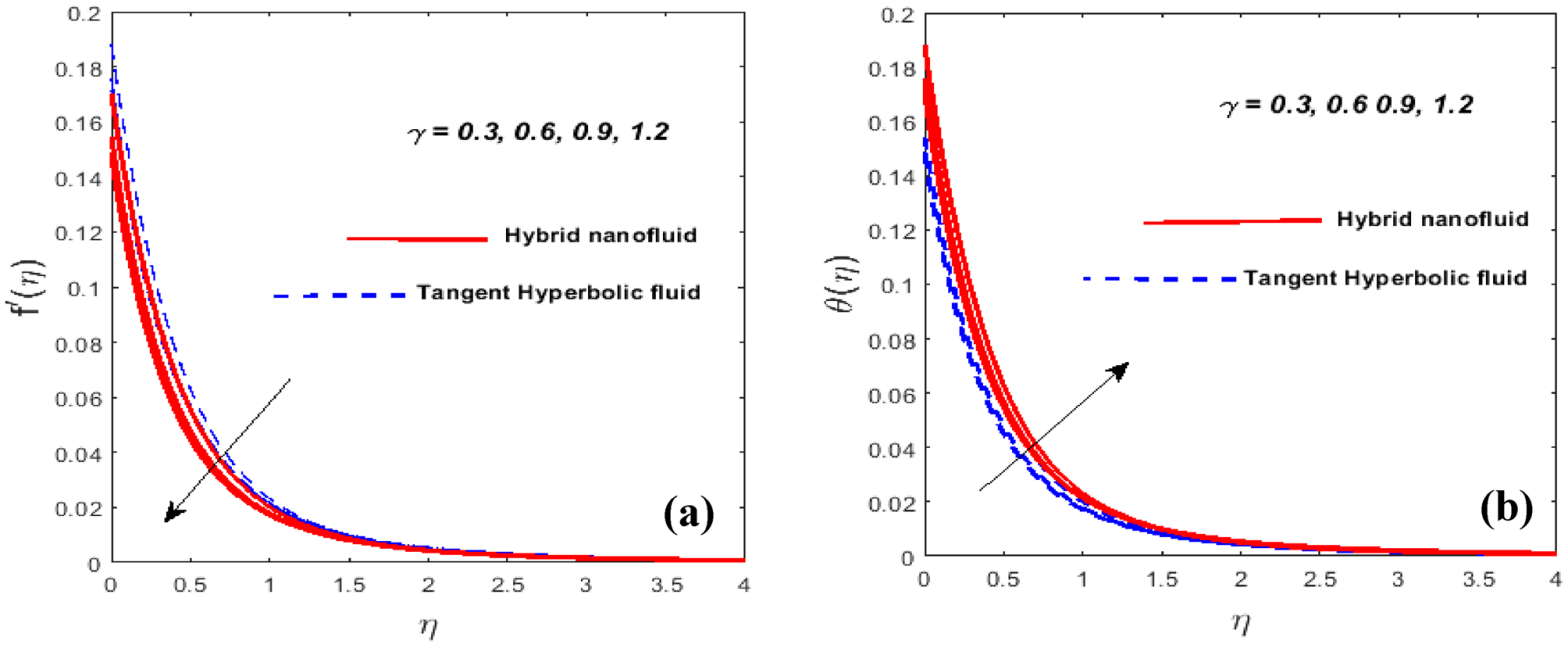
$$\gamma$$ against $$f^{\prime}\left( \eta \right)$$ (**a**) and $$\theta \left( \eta \right)$$ (**b**).

**Figure 5:**
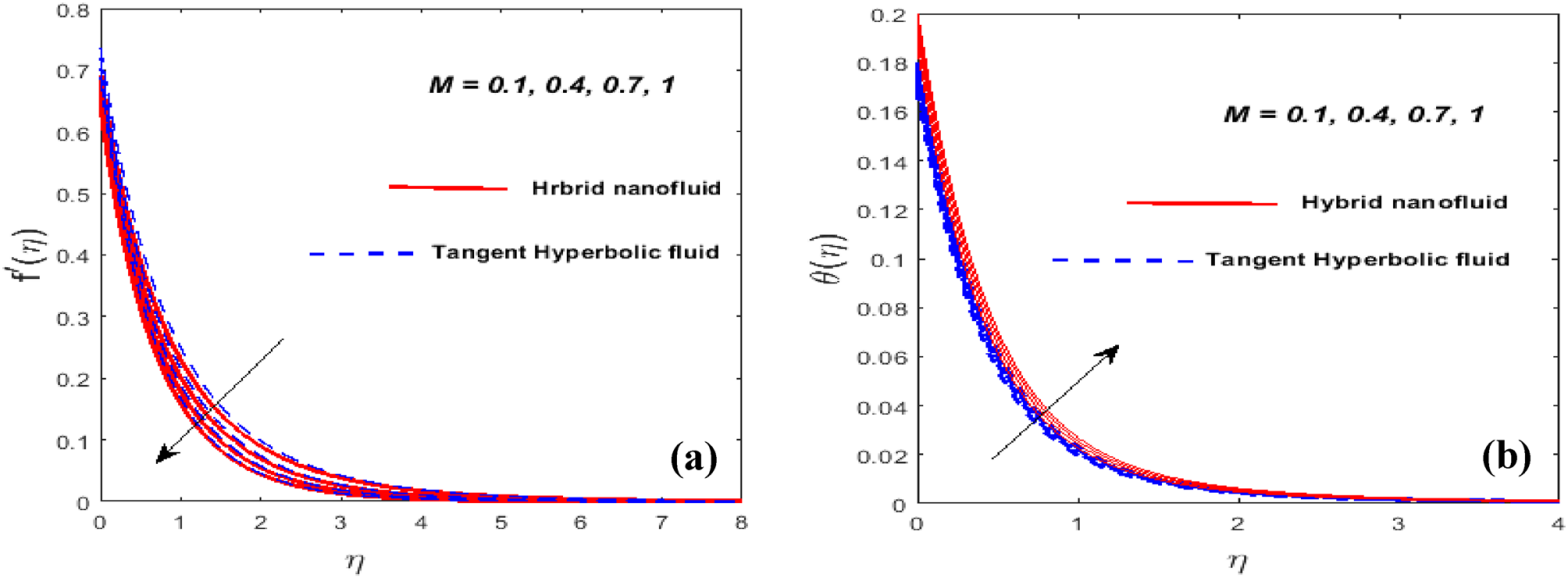
$$M$$ against $$f^{\prime}\left( \eta \right)$$ (**a**) and $$\theta \left( \eta \right)$$ (**b**).

**Figure 6:**
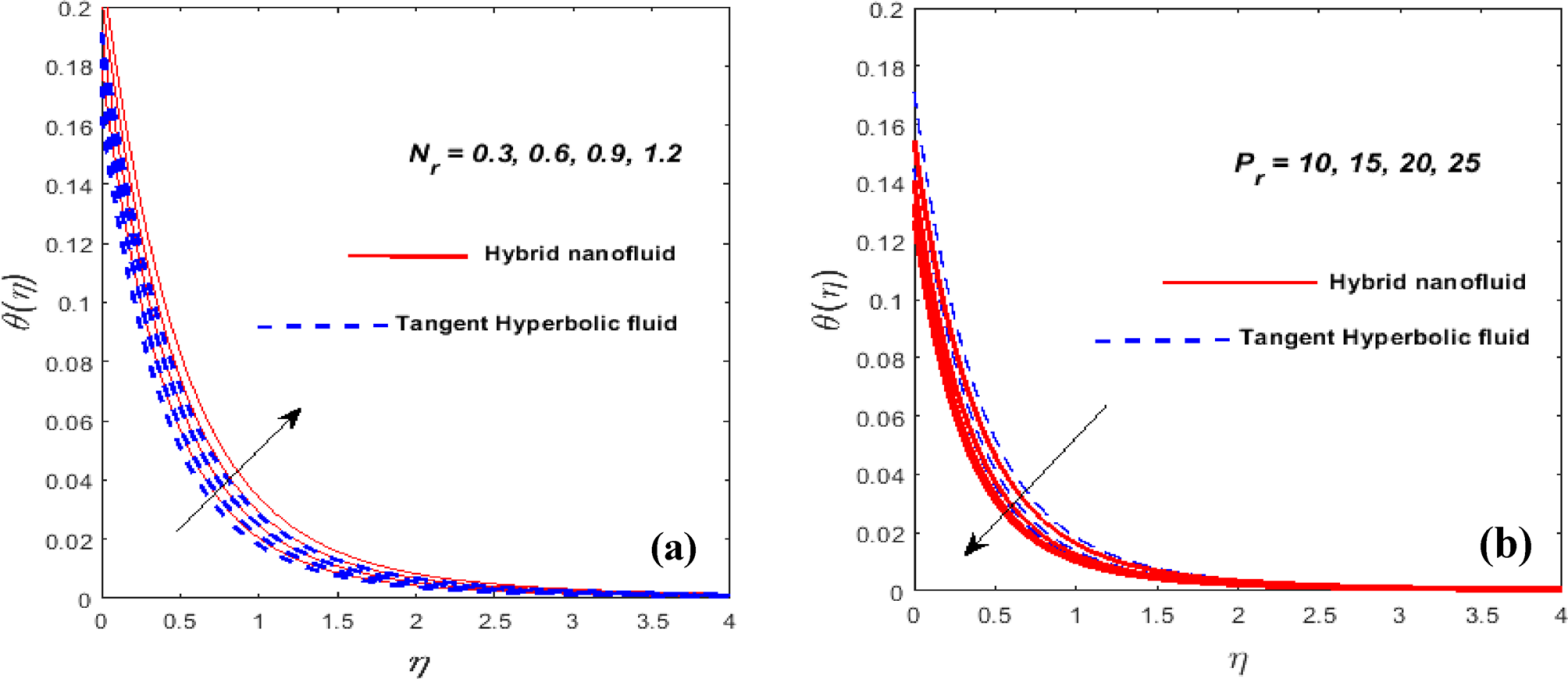
$$N_{r}$$ and $$P_{r}$$ against $$\theta \left( \eta \right).$$

Figure 3a,b outlines the consequence of unsteady parameter $\left( \beta \right)$ on the velocity $\left( {f^{\prime}\left( \eta \right)} \right)$ and temperature $\left( {\theta \left( \eta \right)} \right)$ gradients. We encountered that when $\beta$ approaches, the velocity actively diminishes while temperature erodes. At the sheet surface, the velocity mindset is due to slip conditions. As we jack up ductility slows down allowing for resistance to fluid motion. Temperature and the grain size of the thermal wave are both seen to be falling functions of $\beta .$ In fact, as $\beta$ increases, so does thermal diffusivity. The involvements of the force of slip parameter $\left( \gamma \right)$ on $f^{\prime}\left( \eta \right)$ and $\theta \left( \eta \right)$ is validated in Fig. 4a,b. We intensify the value of, the momentum BL gains, while the velocity is declined. This consequence happens because the disturbance caused by the fluid's velocity being reduced because of frictional smothering between the fluid's droplets and the bottom is partially transferred to the stretching velocity. It appears that the significance of temperature is increasing at a rapid rate. This arises as a byproduct of a change in velocity slip, which slows down the working fluids and increases the thermal conductivity. The involvements of the magnetic parameter $\left( M \right)$ on the $f^{\prime}\left( \eta \right)$ and $\theta \left( \eta \right)$ are visualized in Fig. 5a,b. Doubling the $M$ certainly lessens both the velocity profile and the cross-sectional area of the BL while temperature increases. The magnetic field exerts a force on Lorentz to evoke resistance to the travel of fluid particles which causes the fluid's velocity minimizes. Magnetic variables, in reality, fixate on the Lorentz force. Higher values $M$ have a high Lorentz force, which inhibits density and raises the fluid temperature. When $M = 0,$ the flow is hydrodynamic, however when *M* > 0, the flow is magnetohydrodynamic. Figure 6a,b exhibits how the temperature profile shapes as the $N_{r}$ and $P_{r}$ are active for a set of values. The method will ensure that as the $N_{r}$ is rose, the temperature went up see Fig. 6a. It comes as a result of an increase in surface heat flow prompted by $N_{r}$, which benchmarks an upswing in the temperature profile. Figure 6b showcases the suitability of $P_{r}$ on the $\theta \left( \eta \right)$. The temperature profile prevents as $P_{r}$ thaws, we said. Temperature and the thermal layer's breadth are both depleting $P_{r}$ functions. In reality, when $P_{r}$ fluctuates, the thermal diffusivity lessens. This leads to a reduction in the energy transfer threshold, which eventually predisposes to a diminution in the area of the thermal layer.

**Figure 7:**
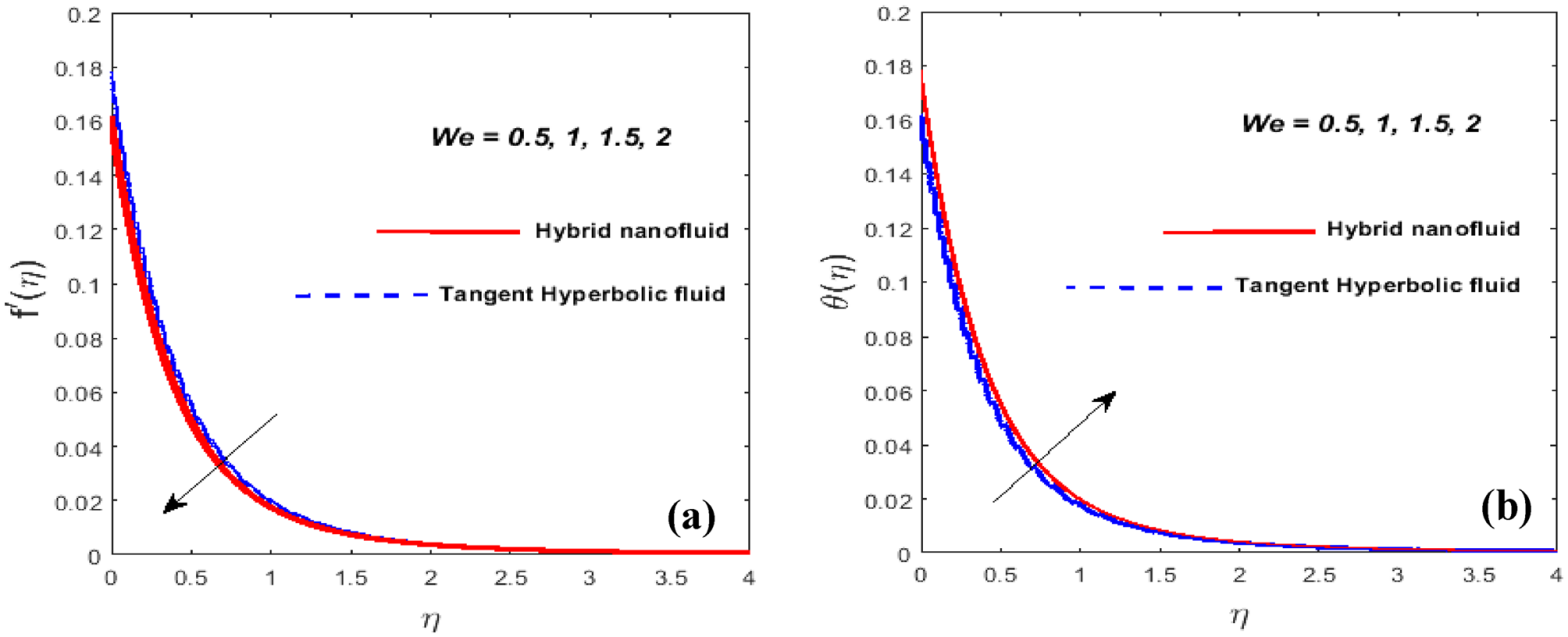
*We* against $f^{\prime}\left( \eta \right)$ (**a**) and $\theta \left( \eta \right)$ (**b**).

The characteristics of the Weissenberg number $\left( {We} \right)$ are responsible for the $f^{\prime}\left( \eta \right)$ and $\theta \left( \eta \right)$ are illustrated in Fig. 7a,b. When $We$ is expanded, the velocity is determined to be decreasing while the fluid's temperature is visible to contribute to the high. The liquid's relaxation time and the specific time process ratio are known as the Weissenberg number. The relaxation speed was adjusted as $We$ kept rising, adding more resistance to the motion of the liquid and, consequently, raising the fluid temperature.

**Figure 8:**
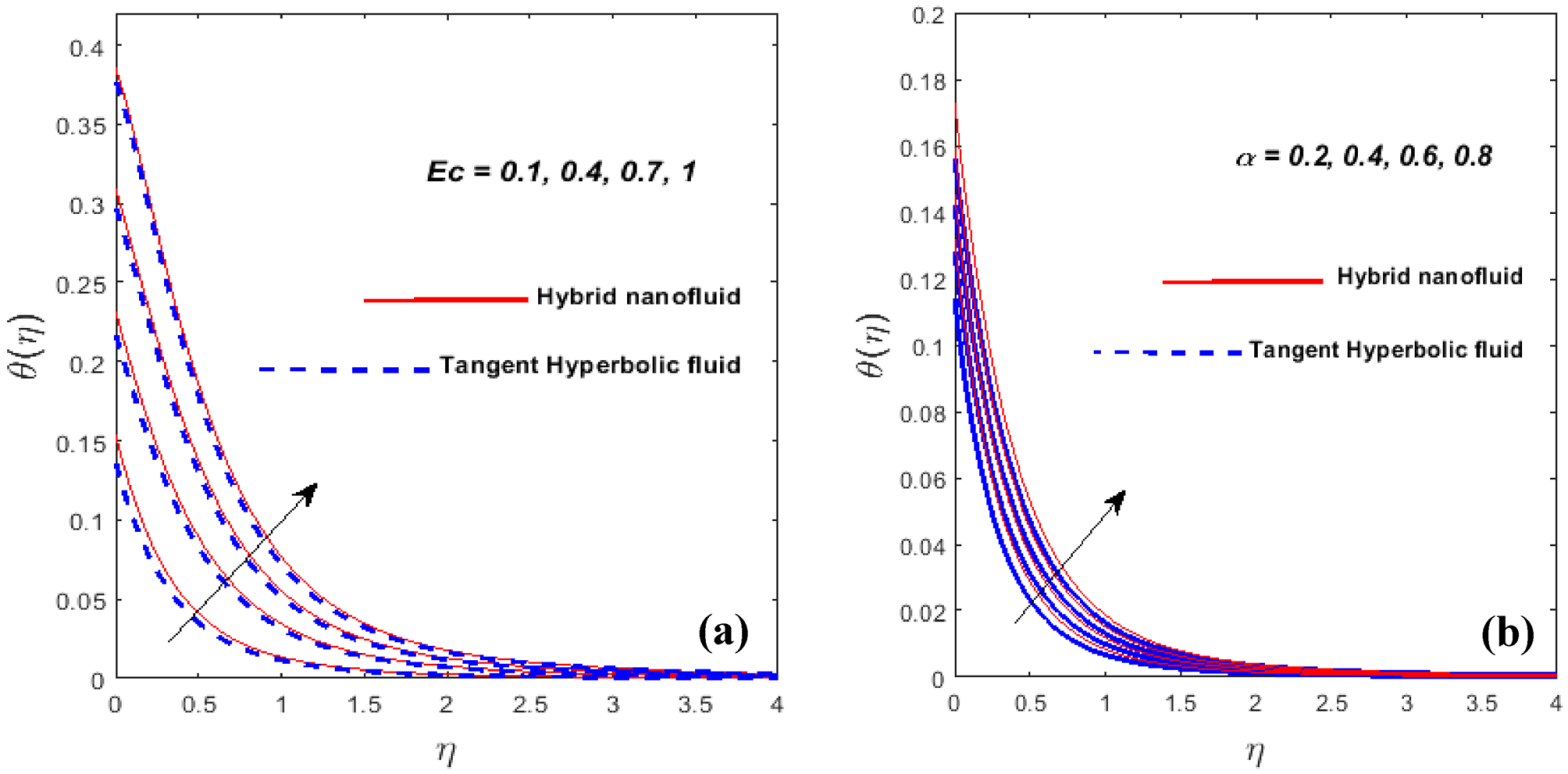
*Ec* and $\alpha$ against $\theta \left( \eta \right).$

Plots of Eckert number (*Ec)* and power law index $\left( \alpha \right)$ on $\theta \left( \eta \right)$ is specified in Fig. 8a,b. It is found that the heat and thermal BL thickness upsurge for larger *Ec*. The heat transfer rate is enhanced as a consequence of the heat energy being stored in the fluid when *Ec* climbs owing to friction forces. Variation of $\theta \left( \eta \right)$ against $\alpha$ is plotted in Fig. 8b Temperature is a steadily rising function of $\alpha$ in this case. Thermal BL thickness is the swelling function of $\alpha .$

**Figure 9:**
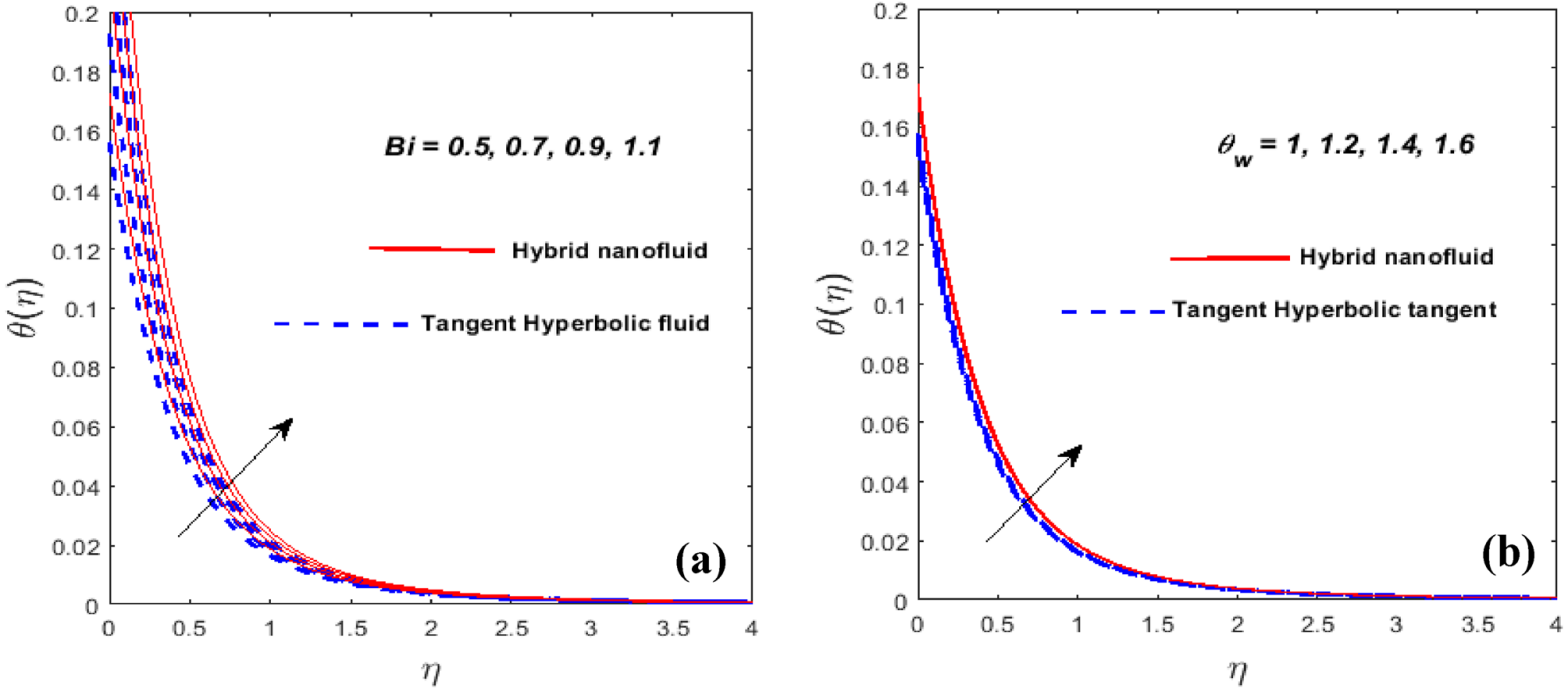
$$Bi$$ and $$\theta_{w}$$ against $$\theta \left( \eta \right).$$

Figure 9a serves to indicate the temperature variation as a factor of Biot number (*Bi*). In general, *Bi* is dictated by the surface's characteristic length, thermal conductivity, and convective heat transfer of the hot fluid under the surface. The constant wall temperature at the surface is regarded by a stronger *Bi*. A higher *Bi* causes the thermal BL to thicken and the heating rate to elevate.

**Figure 10:**
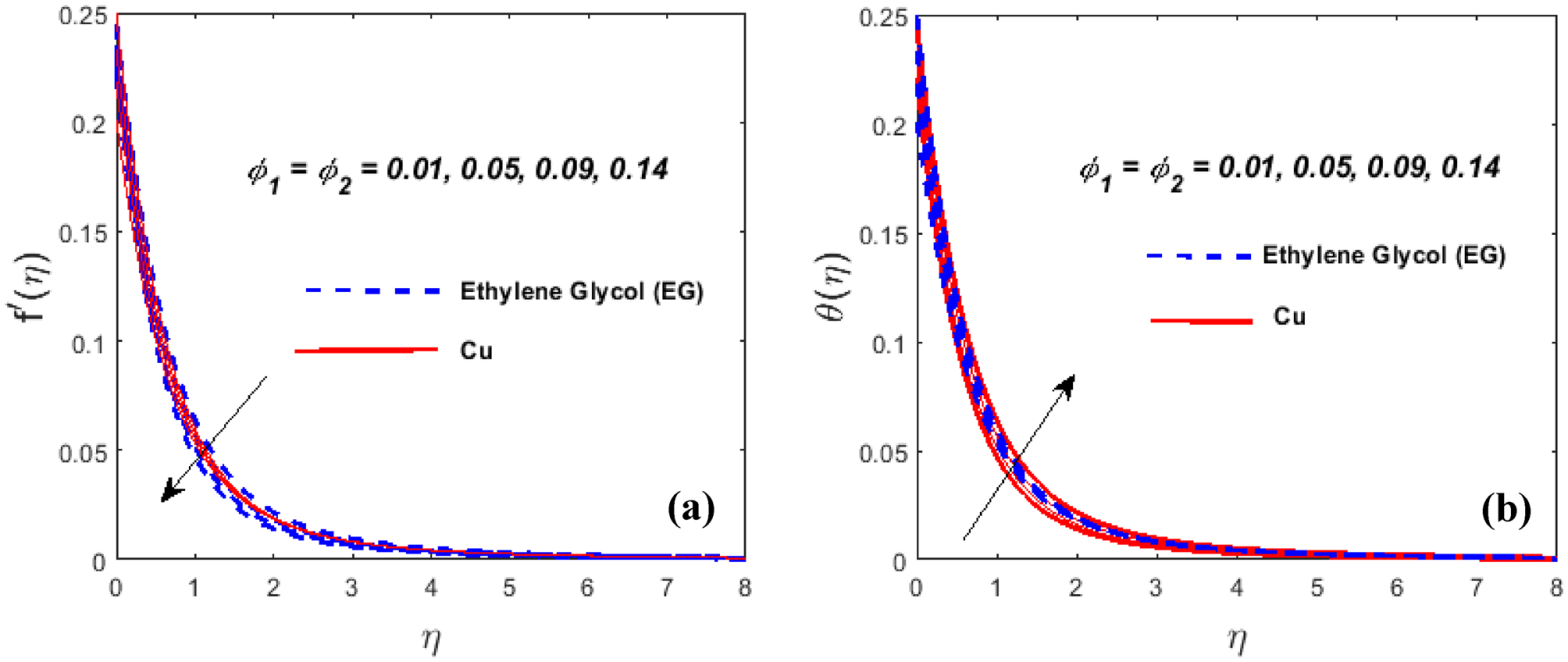
$$\phi_{1\,} \,\,{\text{and}}\,\,\,\phi_{2}$$ against $$f^{\prime}\left( \eta \right)$$ (**a**) and $$\theta \left( \eta \right)$$ (**b**).

Figure 9b demonstrates the influence of $\theta_{w}$ on the $\theta \left( \eta \right).$ For the higher values of $\theta_{w}$ is the increment in thermal layer thickness. Figure 10a,b highlights the impression of the volume fraction of nanoparticles $\left( {\phi_{1\,} \,\,{\text{and}}\,\,\,\phi_{2} } \right)$ on the $f^{\prime}\left( \eta \right)$ and $\theta \left( \eta \right)$. It is noticeable that the velocity field falls as $\phi_{1} \,\,{\text{and}}\,\,\phi_{2}$ grow while the hybrid nanofluid's temperature field expands. Physically, the momentum and thermal BL get denser at the larger volume fraction $\phi_{1} \,\,{\text{and}}\,\,\phi_{2}$. The fractional nanoparticle size in the base fluid appears to have an impact on the effectiveness of the nanofluid and hybrid variants. Because of the increased load, nanoparticles with a higher good fractional range have a lower flowability. Due to the shear-thinning characteristic's dependence on temperature, this phenomenon occurs. Nanoparticles have a high thermal conductivity and transfer rate under a variety of physical conditions, which is a well-known property. Additionally, it is mentioned that the particles' viscosity will decline at higher temperatures.

**Figure 11:**
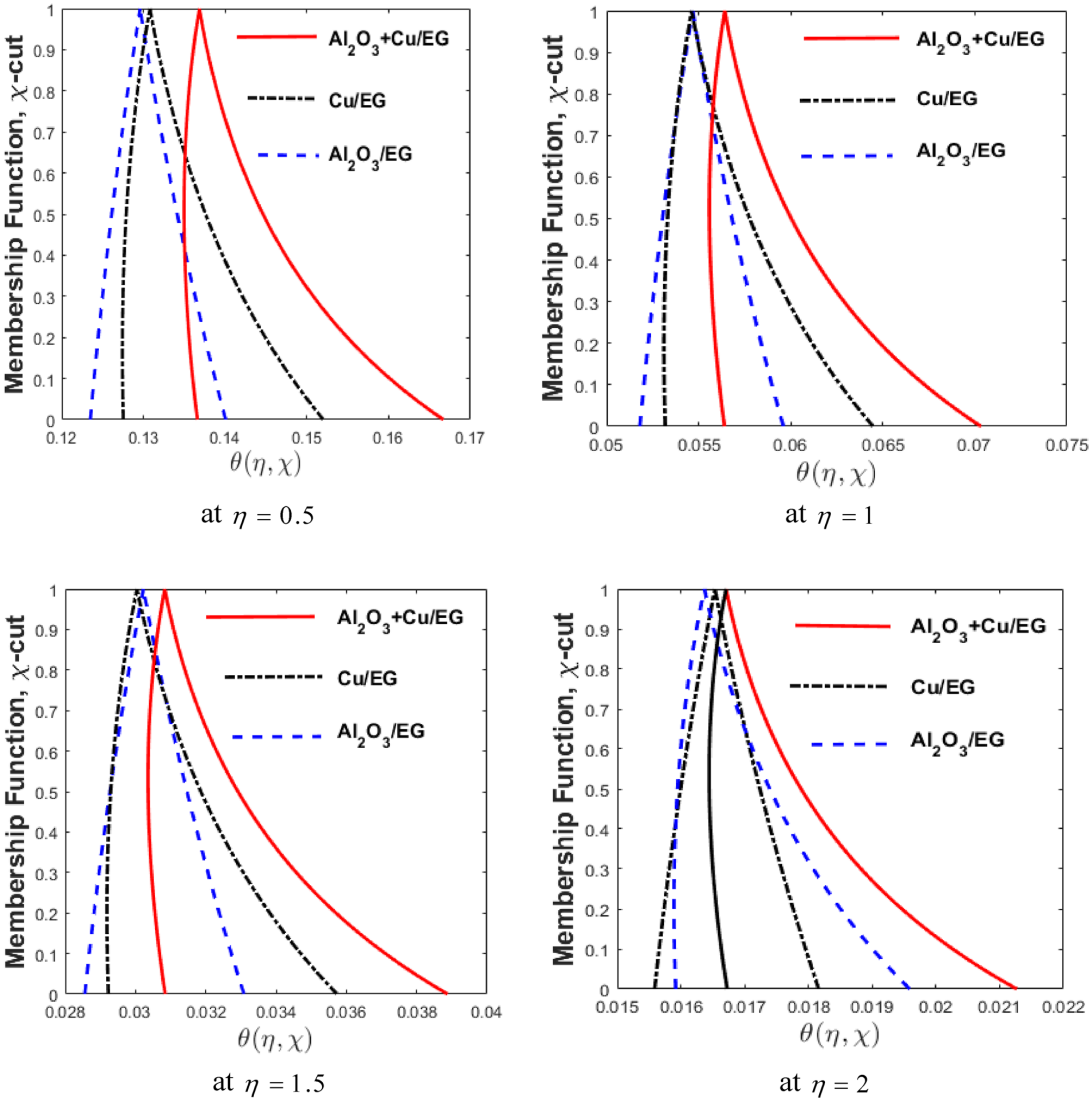
Comparison of ${\text{Al}}_{{2}} {\text{O}}_{{3}} {\text{/EG}}$, ${\text{Cu/EG}}$ and ${\text{Al}}_{{2}} {\text{O}}_{{3}} {\text{ + Cu/EG}}$ hybrid nanofluid for varying of η.

The impact of nanofluids $${\text{Al}}_{{2}} {\text{O}}_{{3}} {\text{/EG}}$$$$\left( {\phi_{1} } \right)$$, $${\text{Cu/EG}}$$$$\left( {\phi_{2} } \right)$$ and hybrid nanofluid $$\left( {{\text{Al}}_{{2}} {\text{O}}_{{3}} {\text{ + Cu/EG}}} \right)$$ on fuzzy temperature profile $$\left( {\theta \left( {\eta ,\,\,\chi } \right)} \right)$$ for distinct values of η are exposed in Fig. 11. We looked at three possible scenarios in these diagrams. Blue-dashed lines signify the situation when $$\phi_{1}$$ is engaged as TFN then $$\phi_{2} = 0.$$ Black lines display the deviation of $$\phi_{2}$$ whereas $$\phi_{1}$$ = 0. In the third scenario, hybrid nanofluid exemplifies through both $$\phi_{1}$$ and $$\phi_{2}$$ being non-zero. Additionally, the $$\theta \left( {\eta ,\,\,\chi } \right)$$ for varied $$\eta$$ is displayed on the horizontal axis, and the MF of the $$\theta \left( {\eta ,\,\,\chi } \right)$$ for varying $$\chi {\text{ - cut}}$$ is displayed on the vertical axis. When especially in comparison to nanofluids $${\text{Al}}_{{2}} {\text{O}}_{{3}} {\text{/EG}}$$ and $${\text{Cu/EG,}}$$ the hybrid nanofluid $$\left( {{\text{Al}}_{{2}} {\text{O}}_{{3}} {\text{ + Cu/EG}}} \right)$$ is found to be better according to fuzzy analysis. A hybrid nanofluid exhibits a more significant heat transfer rate than the other two cases. Physically, hybrid nanofluid is constructed by combining the combined thermal conductivities of $${\text{Al}}_{{2}} {\text{O}}_{{3}}$$ and $${\text{Cu}}$$ to allow for the fastest possible heat transfer. Since $${\text{Cu/EG}}$$ thermal conductivity is higher than $${\text{Al}}_{{2}} {\text{O}}_{{3}} {\text{/EG}}$$, it exhibits a faster rate of heat transfer when compared to $${\text{Al}}_{{2}} {\text{O}}_{{3}} {\text{/EG}}$$ nanofluid.

The numerical variations of the drag coefficient and Nusselt number are depicted in Tables 4 and 5. Table 4 demonstrates the magnitude of growth in relationship to escalating $$Ec,$$$$\beta$$ and $$M$$ estimations while higher $$We,$$ $$\alpha$$ and $$\gamma$$ are associated with the opposite tendency. In Table 5, it is clear that the amplitude of declines in response to higher $$Ec,$$ $$M,$$ *H*, and $$\theta_{w}$$ whereas it grows through growing levels of $$Bi,$$ $$N_{r}$$ and $$We.$$ The thermal efficiency of the liquid can be boosted more efficiently by using a dynamically hybrid nanofluid. Due to the steady dispersion of these two different types of nanoparticles in a single base fluid, the thermal performance improves.

**Table 4:** The values of $Cf_{x}$ for various parameters.

*M*	*Ec*	$\beta$	*We*	$\alpha$	$\gamma$	Tangent Hyperbolic fluid when $\phi_{1} = \phi_{2} = 0.\,\,$	Hybrid nanofluid
0						− 0.259987	− 0.292578
0.1						− 0.264822	− 0.294962
0.2						− 0.269223	− 0.297215
	0					− 0.259987	− 0.294963
	0.2					− 0.269223	− 0.296761
	0.3					− 0.269223	− 0.295861
		0.3				− 0.272315	− 0.294962
		0.4				− 0.275237	− 0.278914
		0.5				− 0.277997	− 0.282003
			0.1			− 0.271435	− 0.270973
			0.2			− 0.270329	− 0.269865
			0.3			− 0.269223	− 0.268756
				0.1		− 0.303861	− 0.304439
				0.2		− 0.267007	− 0.268756
				0.3		− 0.230113	− 0.2330533
					1.7	− 0.247951	− 0.2447321
					1.8	− 0.238683	− 0.2408249
					1.9	− 0.230113	− 0.2310564

**Table 5:** The values of $Nu_{x}$ for various parameters.

*M*	$P_{r}$	*Ec*	*H*	$\beta$	$N_{r}$	$\theta_{w}$	*We*	$B_{i}$	Tangent Hyperbolic fluid $\phi_{1} = \phi_{2} = 0.\,\,$	Hybrid nanofluid
0									0.785601	0.729011
0.1									0.770504	0.717961
0.2									0.759577	0.706968
	0.1								0.166851	0.310356
	0.2								0.201496	0.394763
	0.3								0.232504	0.451746
		0							0.414225	0.395362
		0.2							0.401346	0.394255
		0.3							0.400791	0.393568
			1						0.557705	0.257363
			1.1						0.469747	0.235204
			1.2						0.401346	0.210213
				0.3					0.175019	0.295538
				0.4					0.182920	0.312800
				0.5					0.190572	0.328624
					0.1				0.156512	0.992663
					0.2				0.156312	1.026455
					0.3				0.156140	1.061267
						1.5			0.166851	2.020392
						1.6			0.157916	2.005458
						1.7			0.149387	1.711321
							0.1		0.166884	0.060835
							0.2		0.166868	0.055078
							0.3		0.166851	0.046242
								0.5	0.168305	0.368573
								0.6	0.173179	0.313823
								0.7	0.176754	0.234421

## Conclusions

Unsteady MHD tangent hyperbolic fuzzy hybrid nanofluid identified with convective surface boundary conditions and nonlinear thermal radiation is explored in this work. With the help of the Bvp4c scheme, the boundary value problem is addressed numerically. The key points from the present analysis are addressed below**:**

- On fluid velocity, the performances of Weissenberg number is show the increasing behaviour and the magnetic parameter has the opposite effect on fluid velocity.
- The fluid temperature is eroded by the Prandtl number, while the Lorentz force and Biot numbers produce a more fluid temperature.
- Skin friction decreases with bigger of $\alpha \,\,\,{\text{and}}\,\,\,We$ but rises with an upsurge in $M.$
- The rate of heat transfer is higher when the Eckert number, Biot number, Prandtl number and thermal radiation are larger.
- The fluid temperature rise as $P_{r}$ and $Ec$ escalation, while the fluid velocity decline when $\beta$ the rise.
- The present findings are validated and found to be in excellent accord with the previous finding.
- Via triangular fuzzy MFs, it is highlighted that hybrid nanofluids are significantly more capable of accelerating thermal efficiency than ${\text{Al}}_{{2}} {\text{O}}_{{3}} {\text{/EG}}$ and ${\text{Cu/EG}}$ nanofluids.
- From this research and the figures, we can determine that the hybrid nanofluid has a significantly higher heat transfer rate than the Tangent hyperbolic fluid.
- The temperature rises as thermal conductivity improves. Because copper nanoparticles have a higher thermal conductivity than other metallic nanoparticles, their insertion produces more heat than that of any other type of nanoparticle.
- The heat transfer rate is enhanced for temperature ratio and nanoparticle volume fractions.

## Data Availability

The data used to support the findings of this study are available from the corresponding author upon request.
